# Exploratory analysis of the potential for advanced diagnostic testing to reduce healthcare expenditures of patients hospitalized with meningitis or encephalitis

**DOI:** 10.1371/journal.pone.0226895

**Published:** 2020-01-15

**Authors:** Brent D. Fulton, David G. Proudman, Hannah A. Sample, Jeffrey M. Gelfand, Charles Y. Chiu, Joseph L. DeRisi, Michael R. Wilson

**Affiliations:** 1 School of Public Health, University of California, Berkeley, Berkeley, California, United States of America; 2 Department of Biochemistry and Biophysics, University of California, San Francisco, San Francisco, California, United States of America; 3 Department of Neurology, Weill Institute for Neurosciences, University of California, San Francisco, San Francisco, California, United States of America; 4 Department of Laboratory Medicine, University of California, San Francisco, San Francisco, California, United States of America; 5 Department of Medicine, University of California, San Francisco, San Francisco, California, United States of America; 6 UCSF-Abbott Viral Diagnostics and Discovery Center, San Francisco, California, United States of America; 7 Chan Zuckerberg Biohub, San Francisco, California, United States of America; University of Malta Faculty of Health Sciences, MALTA

## Abstract

**Objective:**

To estimate healthcare expenditures that could be impacted by advanced diagnostic testing for patients hospitalized with meningitis or encephalitis

**Methods:**

Patients hospitalized with meningitis (N = 23,933) or encephalitis (N = 7,858) in the U.S. were identified in the 2010–2014 Truven Health MarketScan Commercial Claims and Encounters Database using ICD-9-CM diagnostic codes. The database included an average of 40.8 million commercially insured enrollees under age 65 per year. Clinical, demographic and healthcare utilization criteria were used to identify patient subgroups early in their episode who were at risk to have high inpatient expenditures. Healthcare expenditures of patients within each subgroup were bifurcated: those expenditures that remained five days after the patient could be classified into the subgroup versus those that had occurred previously.

**Results:**

The hospitalization episode rate per 100,000 enrollee-years for meningitis was 13.0 (95% CI: 12.9–13.2) and for encephalitis was 4.3 (95% CI: 4.2–4.4), with mean inpatient expenditures of $36,891 (SD = $92,636) and $60,181 (SD = $130,276), respectively. If advanced diagnostic testing had been administered on the day that a patient could be classified into a subgroup, then a test with a five-day turnaround time could impact the following mean inpatient expenditures that remained by subgroup for patients with meningitis or encephalitis, respectively: had a neurosurgical procedure ($83,337 and $56,020), had an ICU stay ($34,221 and $46,051), had HIV-1 infection or a previous organ transplant ($37,702 and $62,222), were age <1 year ($35,371 and $52,812), or had a hospital length of stay >2 days ($18,325 and $30,244).

**Discussion:**

Inpatient expenditures for patients hospitalized with meningitis or encephalitis were substantial and varied widely. Patient subgroups who had high healthcare expenditures could be identified early in their stay, raising the potential for advanced diagnostic testing to lower these expenditures.

## Introduction

Meningitis and encephalitis are serious, sometimes life-threatening conditions. The hospitalization rate for meningitis in the United States in 2006 was 24.1 per 100,000 persons [1], whereas the hospitalization rate for encephalitis was estimated in three studies to be approximately 7 per 100,000 persons: 1988–1997 (7.3) [2], 2000–2010 (7.3) [3], 1998–2010 (6.9) [4]. Previous studies have utilized the National Inpatient Sample to estimate the hospital facility costs and charges of patients hospitalized with meningitis or encephalitis. For patients with meningitis, one study estimated the mean facility cost per hospitalization to be $17,100 in 2006 [1]. For patients with encephalitis, another study estimated the median facility charge to be $48,852 in 2010 [4].

Depending on the reimbursement method, hospital charges can be influenced by length of stay (LOS), which can vary significantly among patients with meningitis or encephalitis. One study estimated the median LOS for patients with encephalitis to be 6 days, with an inter-quartile range (IQR) of 3–13 days [4]. Another study estimated the median LOS for patients with meningitis or encephalitis to be 4 days, with median LOS by etiology ranging from 3 to 13 days [5].

One contributor to the varying LOS in this patient population is the difficulty of identifying the underlying disease etiology, potentially leading to delays in appropriate treatment [6]. Several molecular advanced diagnostic tests with culture-independent, broad-detection capability are being deployed that can simultaneously test for a wide range of pathogens, including multiplex pathogen-specific PCR [7], 16S ribosomal RNA sequencing [8], and metagenomic next-generation sequencing (mNGS) [9–11]. These advanced diagnostic tests may contribute to improved patient outcomes and reduced healthcare utilization and expenditures, particularly for patients for whom targeted therapy can be initiated more quickly.

In particular, mNGS is an emerging approach to infectious disease diagnosis that leverages high-throughput, deep sequencing of extracted nucleic acid (RNA and/or DNA) from clinical samples to detect nearly all pathogens in a sample, including viruses, bacteria, fungi, and parasites [9]. Because mNGS does not rely on the use of targeted primers or probes, it has the potential to supplement or even replace conventional serial diagnostic testing based on a physician’s differential diagnosis of the most likely pathogens responsible for the patient’s infectious syndrome. Obstacles to widespread adoption of mNGS include concerns about high costs, long turnaround time, and need for additional data about test performance characteristics. Thus, mNGS is often reserved for use as a late-stage option in diagnostically challenging cases [11–16]. Ongoing clinical validation of mNGS testing for a number of syndromes, however, including meningitis and encephalitis [10], sepsis [17], and pneumonia [10], suggest that this diagnostic technique may soon be available for routine diagnosis of infections. It is imperative that clinical-outcomes and health-economics studies be conducted to determine the clinical scenarios under which mNGS and other advanced diagnostic testing would benefit patients and healthcare payers.

Thus, we used a large source of commercial health insurance claims to accomplish the following three objectives: (1) estimate healthcare expenditures for different subgroups of patients hospitalized with meningitis or encephalitis; (2) describe the timing of healthcare expenditures in meningitis and encephalitis patient subgroups with the aim of estimating healthcare expenditures that might be reduced by the institution of earlier advanced diagnostic testing; and (3) conduct a breakeven analysis to show the required percentage reduction of healthcare expenditures that remained five days after the administration of an advanced diagnostic test by varying the probability of the test changing care. To our knowledge, this is the first study to estimate healthcare expenditures for patients hospitalized with meningitis and encephalitis based on actual amounts paid instead of charges, which are usually significantly higher [18, 19].

## Methods

### Study participants

This study includes enrollees from the Truven Health MarketScan Commercial Claims and Encounters Database, which is a U.S. database of healthcare claims of insured employees, most of whom are employed by large employers, and their dependents. The database is demographically representative of the U.S. population under age 65 and is representative of their types of health plans (see Table 1) [20, 21]. Since 1990, the database has been used in over 1,100 studies published in peer-reviewed journals [22]. The database includes healthcare claims and encounter data for professional services, inpatient and outpatient facilities, and prescription drugs as well as enrollee demographic information, such as gender, age, and the census region. The healthcare claims are based on actual amounts paid by the insurer and cost sharing paid by the enrollee. We had access to the 2010 to 2014 data, resulting in a five-year study period from January 1, 2010 to December 31, 2014, which included an average of 40.8 million enrollees less than 65 years old per year. To avoid missing healthcare utilization associated with a meningitis or encephalitis episode, we excluded diagnoses that occurred during the first 90 days of 2010 or the last 90 days of 2014; 90 days represents the 98^th^ percentile for an episode duration. To adjust for inflation, we inflated expenditures to 2014 dollars using the Consumer Price Index.

**Table 1:** Demographic characteristics and episode incidence rates of enrollees hospitalized with meningitis or encephalitis, 2010 to 2014.

	Enrollees		Meningitis Episodes				Encephalitis Episodes			
Characteristic	No. per Year (mean)	Per-cent	No.	Rate	Rate (95% CI)		No.	Rate	Rate (95% CI)	
Total	40,831,909	100%	23,933	13.0	12.9	13.2	7,858	4.3	4.2	4.4
Gender										
Male	19,856,810	49%	11,485	12.9	12.6	13.1	3,801	4.3	4.1	4.4
Female	20,975,100	51%	12,448	13.2	13.0	13.4	4,057	4.3	4.2	4.4
Age (years)										
<1	618,226	2%	2,836	101.9	98.2	105.7	174	6.3	5.3	7.2
1–4	1,937,447	5%	488	5.6	5.1	6.1	345	4.0	3.5	4.4
5–19	9,018,190	22%	3,193	7.9	7.6	8.1	1,383	3.4	3.2	3.6
20–44	15,258,192	37%	9,499	13.8	13.6	14.1	2,308	3.4	3.2	3.5
45–64	13,999,855	34%	7,917	12.6	12.3	12.8	3,648	5.8	5.6	6.0
U.S. Census Region										
Northeast	7,478,604	18%	4,336	12.9	12.5	13.3	1,606	4.8	4.5	5.0
Midwest	9,349,158	23%	5,276	12.5	12.2	12.9	1,714	4.1	3.9	4.3
South	14,735,551	36%	9,297	14.0	13.7	14.3	2,905	4.4	4.2	4.5
West	8,400,686	21%	4,464	11.8	11.5	12.2	1,432	3.8	3.6	4.0
Unknown	867,910	2%	560	14.3	13.2	15.5	201	5.1	4.4	5.9
Residence										
Urban	34,277,582	84%	20,310	13.2	13.0	13.3	6,603	4.3	4.2	4.4
Rural	5,702,434	14%	3,065	11.9	11.5	12.4	1,057	4.1	3.9	4.4
Unknown	851,893	2%	558	14.6	13.3	15.8	198	5.2	4.4	5.9
Plan type										
PPO	23,374,941	57%	14,774	14.0	13.8	14.3	4,952	4.7	4.6	4.8
POS	2,329,875	6%	1,563	14.9	14.2	15.6	505	4.8	4.4	5.2
EPO	872,104	2%	613	15.6	14.4	16.9	183	4.7	4.0	5.3
HMO	4,537,299	11%	3,077	15.1	14.5	15.6	899	4.4	4.1	4.7
Other	4,480,940	11%	2,576	12.8	12.3	13.3	843	4.2	3.9	4.5
Unknown	5,236,750	13%	1,330	5.6	5.3	5.9	476	2.0	1.8	2.2

Notes: The first 90 days and the last 90 days are excluded from the 2010 to 2014 period. The total number of enrollee-months of 2,204,923,108 equals the mean number of enrollees per year multiplied by 4.5 years multiplied by 12 months. Rate is per 100,000 enrollee-years. No.: number, CI: confidence interval, PPO: preferred provider organization plan, POS: point of service plan, EPO: exclusive provider organization plan, HMO: health maintenance organization plan. Other Plan Type includes comprehensive, consumer-driven health plan (CDHP), high-deductible health plan (HDHP), and point-of-service (POS) capitation.

Source: Authors’ analysis of 2010 to 2014 Truven Health MarketScan Commercial Claims and Encounters Database

To identify hospitalized patients with meningitis or encephalitis, we selected ICD-9-CM diagnostic codes based on previous studies of meningitis [1, 23, 24] and encephalitis [4]; the included diagnostic codes are listed in Tables A and B in S1 Appendix, respectively. Consistent with those studies, we included patients with these codes in either the principal or any of the 13 secondary diagnosis fields. Because some patients had both a meningitis and encephalitis diagnosis, we coded two types of hospitalized patients based on clinical phenotype as follows: (1) patients with a meningitis diagnosis who did not have an encephalitis diagnosis (N = 23,933), and (2) patients with an encephalitis diagnosis who may have had a meningitis diagnosis (N = 7,858). We did not classify patients by the etiology of their neuroinflammatory condition because the ICD-9-CM diagnostic codes do not represent the full spectrum of meningitis and encephalitis etiologies, and we could not clinically adjudicate individual cases.

In order to estimate healthcare expenditures that could be impacted by advanced diagnostic testing for patients hospitalized with meningitis or encephalitis, we defined the following five subgroups of hospitalized patients *a priori* that spanned from an inclusive subgroup of patients who had a hospital LOS > 2 days to relatively exclusive, higher-cost subgroups based on widely recognized, high-risk clinical, demographic and healthcare utilization criteria gleaned from the medical literature [3, 4, 25–27]: Subgroup 1: patients who had a LOS greater than two days (N = 16,226 meningitis and N = 6,468 encephalitis); Subgroup 2: patients who were admitted to an intensive care unit (ICU) any time during a hospital stay (N = 5,894 meningitis and N = 3,430 encephalitis); Subgroup 3: patients who received a neurosurgical procedure, which was most commonly related to neuromonitoring and shunting for meningitis and neuromonitoring and biopsy for encephalitis (see CPT codes in Tables C and D in S1 Appendix, respectively) (N = 1,233 meningitis and N = 535 encephalitis); Subgroup 4: patients who had a diagnosis code indicating that they had HIV-1 infection or a previous organ transplant (N = 513 meningitis and N = 289 encephalitis); and Subgroup 5: patients who were less than one year old at the time of admission (N = 2,835 meningitis and N = 174 encephalitis). Patients could be classified into more than one subgroup because the subgroups were not mutually exclusive. Table E in S1 Appendix reports the patient counts among the subgroups.

### Statistical analysis

We were interested in the degree to which these subgroup characteristics predicted high healthcare expenditures, in part, because these criteria could be evaluated early in a patient’s stay. For example, 82% of patients with encephalitis who were admitted to the ICU were admitted on the first day of their episode. Hence, if an advanced diagnostic test had been ordered upon the ICU admission, it would probably not have prevented that ICU admission, but may have decreased its length. Similarly, an advanced diagnostic test would not necessarily avoid the need for many neurosurgical procedures, but the neurosurgical procedure serves as a predictor for an unknown etiology and high healthcare expenditures. Furthermore, because our goal was to evaluate demographic and clinical characteristics that predicted which patients would be high cost, we did not estimate a healthcare-expenditure regression model controlling for those characteristics, which would have estimated the independent association between each characteristic and healthcare expenditures.

The unit of analysis was chosen to be an episode of care for a patient hospitalized with meningitis or encephalitis. A patient who had two or more hospitalizations with meningitis (or encephalitis) during a 180-day period was counted as one episode (which was the case for 16% of the meningitis episodes and 6% of the encephalitis episodes) because an advanced diagnostic test given in an earlier hospitalization may reduce the number or lengths of stay of subsequent hospitalizations. Thus, our hospital measures (e.g., LOS and inpatient expenditures) were added across all hospitalizations within an episode. The 180-day follow-up period was designed to capture post-discharge expenditures, particularly hospital readmissions, as survivors of meningitis or encephalitis can have a number of ongoing medical complications in the weeks and months after their more acute illness (e.g., seizures, increased intracranial pressure, strokes, and a variety of medical complications). If a subsequent hospitalization with a meningitis or encephalitis diagnostic code occurred after the 180-day period from the initial diagnosis, it was considered to be a separate episode.

Inpatient healthcare utilization measures included hospitalizations, physician services, prescription drugs, diagnostic tests and rehabilitation, while outpatient healthcare utilization measures included hospital services, physician visits, physical therapy and prescription drugs. To include nearly all healthcare utilization and expenditures related to the episode, a 30-day look-back period prior to the hospitalization was examined to capture healthcare utilization that had meningitis or encephalitis diagnosis codes. Given that it was not possible to reliably classify whether prescription drugs were related to the episode, only a seven-day look-back period was used for prescription drugs, and all subsequent prescription drug utilization during the hospitalization was assumed to be related to the episode. A 180-day period following hospital discharge was also examined to capture healthcare utilization that had meningitis or encephalitis diagnosis codes. Again, as it was not possible to reliably classify prescription drugs related to the episode, only a 30-day post-discharge period was used, and all prescription drug utilization was assumed to be related to the episode.

The healthcare expenditure measure included the amount paid by the insurer plus the amount paid by the patient via cost sharing. Because inpatient expenditures accounted for an average of 97% of total expenditures related to an episode, most of our analyses focused on these expenditures. Inpatient expenditures included both inpatient hospital and inpatient rehabilitation, but the latter type only accounted for an average of 1% of total inpatient expenditures. When only inpatient expenditures were considered, the beginning of an episode was defined as the initial hospital admission day. For a given hospitalization, the general floor facility expenditure was reported in a single healthcare claim; therefore, we spread this expenditure evenly across a patient’s length of stay in the hospital. For patients who had two or more hospitalizations during an episode, inpatient hospital expenditures were coded as zero between the hospitalizations.

We estimated the healthcare expenditures remaining for an episode after a subgroup-defining event. There is a lack of evidence on the share of patients whose care would be impacted by the results of an advanced diagnostic test, and for those impacted, there is also a lack of evidence on the degree to which their care would be impacted. Therefore, we conducted a break-even simulation analysis by varying the share of patients whose care would be impacted as well as the degree of the impact. We assumed an advanced diagnostic test cost $2,000 and calculated the breakeven percentage reduction (δ) of remaining healthcare expenditures five days after the sub-group defining event for patients hospitalized with meningitis or encephalitis, based on Eq 1: *HCE*_*test*_ is healthcare expenditures of the test ($2,000), *HCE*_*0*_ is healthcare expenditures without the test, and *p(change care)* is the probability that the test impacts care. The breakeven percentage reduction (δ) is calculated for each of the five patient subgroups, which have different estimated mean expenditures remaining (*HCE*_*0*_), and the calculation allows the probability of the test changing care to range from 1% to 20%, based on the share of patients’ care being affected by an advanced diagnostic test in a previous study, while accounting for the delay in administering the test and the evolving nature of these tests [28].

$$\delta =\frac{{HCE}_{test}}{{HCE}_{0}\times p(change\;care)}$$ (1)

The Truven database is de-identified; therefore, the study did not require approval by an institutional review board because it was not considered human subjects research.

## Results

Table 1 shows the demographic characteristics for all enrollees and for patients hospitalized with meningitis or encephalitis from 2010 to 2014 (excluding the first 90 days and the last 90 days of the period). During this period, the sample included 2,204,923,108 enrollee-months, averaging 40.8 million enrollees per year who had a total of 23,933 episodes with meningitis and 7,858 episodes with encephalitis involving at least one hospitalization. The episode rate per 100,000 enrollee-years for meningitis was 13.0 (95% CI: 12.9 to 13.2) and for encephalitis was 4.3 (95% CI: 4.2 to 4.4), which were lower than previous studies because 16% of the meningitis and 6% of the encephalitis episodes involved multiple hospitalizations and because our sample was restricted to the insured population under age 65, which is healthier than the general population [1–4]. These rates were similar for females and males. For meningitis, the rate for the very young (less than one year old) was extremely high at 101.9 (95% CI: 98.2 to 105.7); and for encephalitis, the rates for the very young (6.3, 95% CI: 5.3 to 7.2) and for adults aged 45 to 64 years (5.8, 95% CI: 5.6 to 6.0) were higher than for other age groups (*p*<0.001).

Table 2 shows healthcare expenditure statistics by type of healthcare utilization for patients hospitalized with meningitis or encephalitis from 2010 to 2014. These statistics are based on all episodes, including episodes that did not incur expenditures for a particular type of healthcare utilization, in order to describe the full distribution of expenditures for each type of healthcare utilization that might be impacted by advanced diagnostic testing. Among the 23,933 meningitis episodes, the mean total healthcare expenditure was $37,904, but among the 7,858 encephalitis episodes, the mean total healthcare expenditure was 64% higher at $62,309. This difference is partially explained by encephalitis episodes having longer hospitalization LOS than meningitis episodes (mean of 13.1 days versus 8.3 days, *p*<0.001) and being more likely to include a stay in the ICU (43.6% versus 24.6%, *p*<0.001). The mean expenditures for inpatient diagnostic tests were $1,145 and $1,569 for meningitis and encephalitis episodes, respectively.

**Table 2:** Healthcare expenditure statistics of patients hospitalized with meningitis or encephalitis, 2010 to 2014.

	Healthcare Expenditure Statistics for All Episodes (1)						
Healthcare Expenditure Type	No. of Episodes Incurring Expend-tures (2)	Mean	Percent-age of Total	Standard Devi-ation	25^th^ Per-centile	50^th^ Per-centile	75^th^ Per-centile
**Meningitis**							
Inpatient Expenditures							
General Floor	23,933	$24,814	65.5%	$67,162	$5,833	$9,760	$19,924
Intensive care unit (ICU) (3)	5,138	$4,804	12.7%	$28,541	$0	$0	$0
Physician Services	22,846	$1,554	4.1%	$2,616	$493	$927	$1,725
Prescription Drugs	17,440	$4,335	11.4%	$15,478	$0	$1,015	$3,230
Diagnostic Tests	14,829	$1,145	3.0%	$3,851	$0	$322	$1,104
Rehabilitation	316	$240	0.6%	$3,249	$0	$0	$0
Subtotal	23,933	$36,891	97.3%	$92,636	$8,779	$14,085	$28,889
Outpatient Expenditures							
Before Hospitalization	5,605	$316	0.8%	$2,998	$0	$0	$0
After Hospitalization							
Outpatient Hospital Visits	3,128	$269	0.7%	$2,452	$0	$0	$0
Physician Visits	7,998	$59	0.2%	$134	$0	$0	$82
Physical Therapy	324	$26	0.1%	$480	$0	$0	$0
Prescription Drugs	13,568	$343	0.9%	$2,292	$0	$12	$148
Subtotal	18,600	$1,013	2.7%	$4,664	$11	$182	$635
Total	23,933	$37,904	100.0%	$93,138	$9,257	$14,761	$30,124
**Encephalitis**							
Inpatient Expenditures							
General Floor	7,826	$36,603	58.7%	$83,864	$8,674	$16,668	$35,686
Intensive care unit (ICU) (3)	3,024	$8,951	14.4%	$36,515	$0	$0	$4,634
Physician Services	7,607	$2,821	4.5%	$5,958	$775	$1,530	$3,034
Prescription Drugs	5,860	$8,675	13.9%	$32,575	$0	$1,403	$5,391
Diagnostic Tests	5,053	$1,569	2.5%	$4,354	$0	$367	$1,464
Rehabilitation	531	$1,562	2.5%	$10,106	$0	$0	$0
Subtotal	7,858	$60,181	96.6%	$130,276	$13,964	$25,649	$54,765
Outpatient Expenditures							
Before Hospitalization	1,043	$297	0.5%	$2,693	$0	$0	$0
After Hospitalization							
Outpatient Hospital Visits	1,608	$942	1.5%	$6,268	$0	$0	$0
Physician Visits	2,332	$78	0.1%	$186	$0	$0	$86
Physical Therapy	456	$226	0.4%	$1,988	$0	$0	$0
Prescription Drugs	4,751	$584	0.9%	$2,537	$0	$36	$314
Subtotal	6,046	$2,128	3.4%	$8,226	$12	$272	$1,263
Total	7,858	$62,309	100%	$132,016	$14,745	$26,868	$56,880

(1) The healthcare expenditure statistics are based on all episodes: 23,933 episodes for meningitis and 7,858 episodes for encephalitis. Hence, the statistics include episodes that did not incur expenditures for a particular type of healthcare utilization (which were coded as zero dollars), in order to describe the full distribution of expenditures for each type.

(2) The number of episodes in this column is the number that incurred expenditures for that type of healthcare utilization.

(3) Intensive care unit (ICU) expenditures could usually be separated from general floor expenditures, which was the case for 5,138 of the 5,894 patients with meningitis who had an ICU stay and for 3,024 of the 3,430 patients with encephalitis who had an ICU stay. For the remaining patients with an ICU stay, their ICU expenditures were included within general floor expenditures.

Notes: The first 90 days and the last 90 days are excluded from the 2010 to 2014 period. Expenditures are reported in 2014 dollars. No.: number.

Source: Authors’ analysis of 2010 to 2014 Truven Health MarketScan Commercial Claims and Encounters Database

Inpatient expenditures for patients hospitalized with meningitis or encephalitis were $36,891 (SD = $92,636) and $60,181 (SD = $130,276), respectively, accounting for 97.3% and 96.6% of the total healthcare expenditures associated with a meningitis or encephalitis episode, respectively. Inpatient expenditures varied widely among episodes, as the coefficient of variation (standard deviation divided by the mean) was 2.5 for patients with meningitis and 2.2 for patients with encephalitis. The distribution of inpatient expenditures by episode was skewed toward high expenditures, as the mean inpatient expenditure exceeded the 75^th^ percentile expenditure for both meningitis and encephalitis episodes.

**Fig 1:**
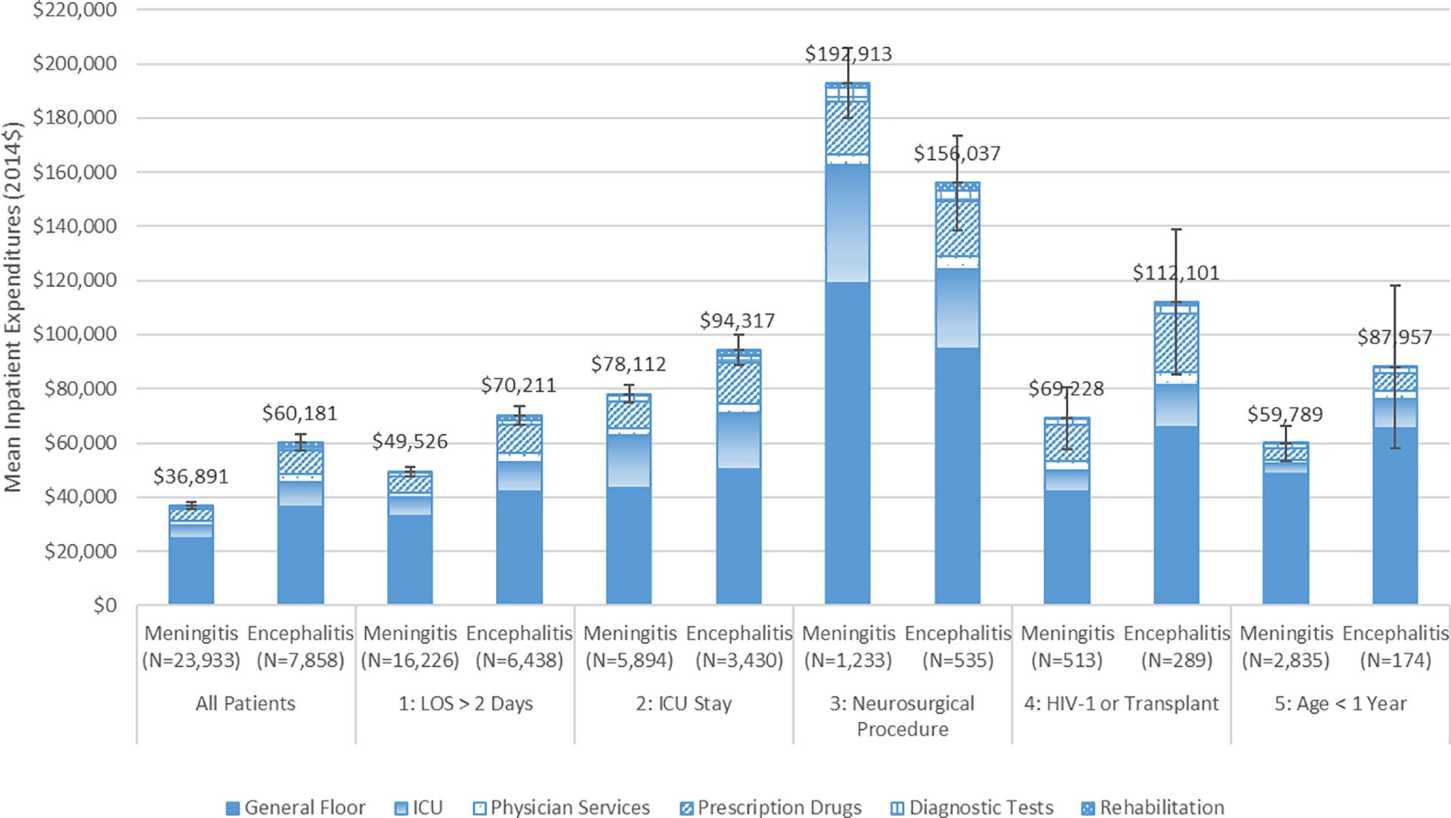
Mean inpatient expenditures by type for hospitalized patient episodes for meningitis or encephalitis by patient subgroup, 2010 to 2014. Notes: LOS: length of stay; IP: inpatient; ICU: intensive care unit The first 90 days and the last 90 days are excluded from the 2010 to 2014 period. Expenditure statistics are based on inpatient expenditures for a patient episode, including re-admissions. Mean expenditures for each type of utilization are calculated using all patient episodes, not just the episodes that had that type of expense. The values reported in the figure are the total mean inpatient expenditures, and the error bars indicate the 95% confidence intervals of these estimated means. Source: Authors’ analysis of 2010 to 2014 Truven Health MarketScan Commercial Claims and Encounters Database.

Fig 1 shows the mean inpatient expenditures for all patients and the five patient subgroups according to the following types of spending: general floor, ICU, physician services, prescription drugs, diagnostic tests and rehabilitation. When including all patients, hospital general floor expenditures accounted for well over half total inpatient expenditures for patients with meningitis (67%) and encephalitis (61%). The next highest shares were for ICU expenditures (13% and 15%, respectively) and prescription drugs (12% and 14%, respectively) for patients with meningitis or encephalitis.

Many of the patients with high inpatient expenditures belonged to one of the patient subgroups that had been defined *a priori*. Patients who received a neurosurgical procedure had the highest mean expenditures among the five patient subgroups: $192,913 for patients with meningitis and $156,037 for patients with encephalitis. As compared to the mean expenditures of all patients with meningitis or encephalitis, patients in the other respective subgroups also had higher mean expenditures: HIV-1 infection or a previous organ transplant ($69,228 and $112,101), had an ICU stay ($78,112 and $94,317), were less than one year old ($59,789 and $87,957), or had a LOS greater than two days ($49,526 and $70,211), respectively.

To formally show how the subgroups perform in predicting high-cost episodes, Table 3 reports the sensitivity, specificity and predictive values of the subgroups predicting that an episode was in the top 25th percentile for spending, which had a threshold of $28,889 and $54,765 for meningitis episodes and encephalitis episodes, respectively. This analysis excluded Subgroup 1 (LOS > 2 days) because that subgroup was not designed to predict high-cost episodes. For meningitis episodes, these subgroups had a high sensitivity (90.4%) and negative predictive value (96.2%). However, because these subgroups were somewhat inclusive, the specificity was 81.5%, and the positive predictive value was only 62.0%, meaning if an episode was in at least one subgroup (excluding subgroup 1), then there was only a 62% chance that the episode was in the top 25% of expenditures for meningitis episodes. For encephalitis episodes, the results were similar. These subgroups had a high sensitivity (99.7%) and negative predictive value (99.2%). However, because these subgroups were somewhat inclusive, the specificity was only 69.7%, and the positive predictive value was only 52.3%, meaning if an episode was in at least one subgroup (excluding subgroup 1), then there was only a 52.3% chance that the episode was in the top 25% of expenditures for encephalitis episodes.

**Table 3:** Sensitivity, specificity and predictive values of subgroups as a predictor for episodes in the top 25^th^ percentile of healthcare expenditures.

Meningitis Episodes	Top 25%	Not in Top 25%	Totals	Predictive Value
In at least one subgroup (except #1)	5,406	3,320	8,726	62.0%
Not in at least one subgroup (except #1)	577	14,630	15,207	96.2%
Totals	5,983	17,950	23,933	
Sensitivity and specificity	90.4%	81.5%		
				
Encephalitis Episodes	Top 25%	Not in Top 25%	Totals	Predictive Value
In at least one subgroup (except #1)	1,959	1,784	3,743	52.3%
Not in at least one subgroup (except #1)	6	4,109	4,115	99.9%
Totals	1,965	5,893	7,858	
Sensitivity and specificity	99.7%	69.7%		

Notes: Subgroup #1: episodes with length of stay > 2 days

Source: Authors’ analysis of 2010 to 2014 Truven Health MarketScan Commercial Claims and Encounters Database

**Fig 2:**
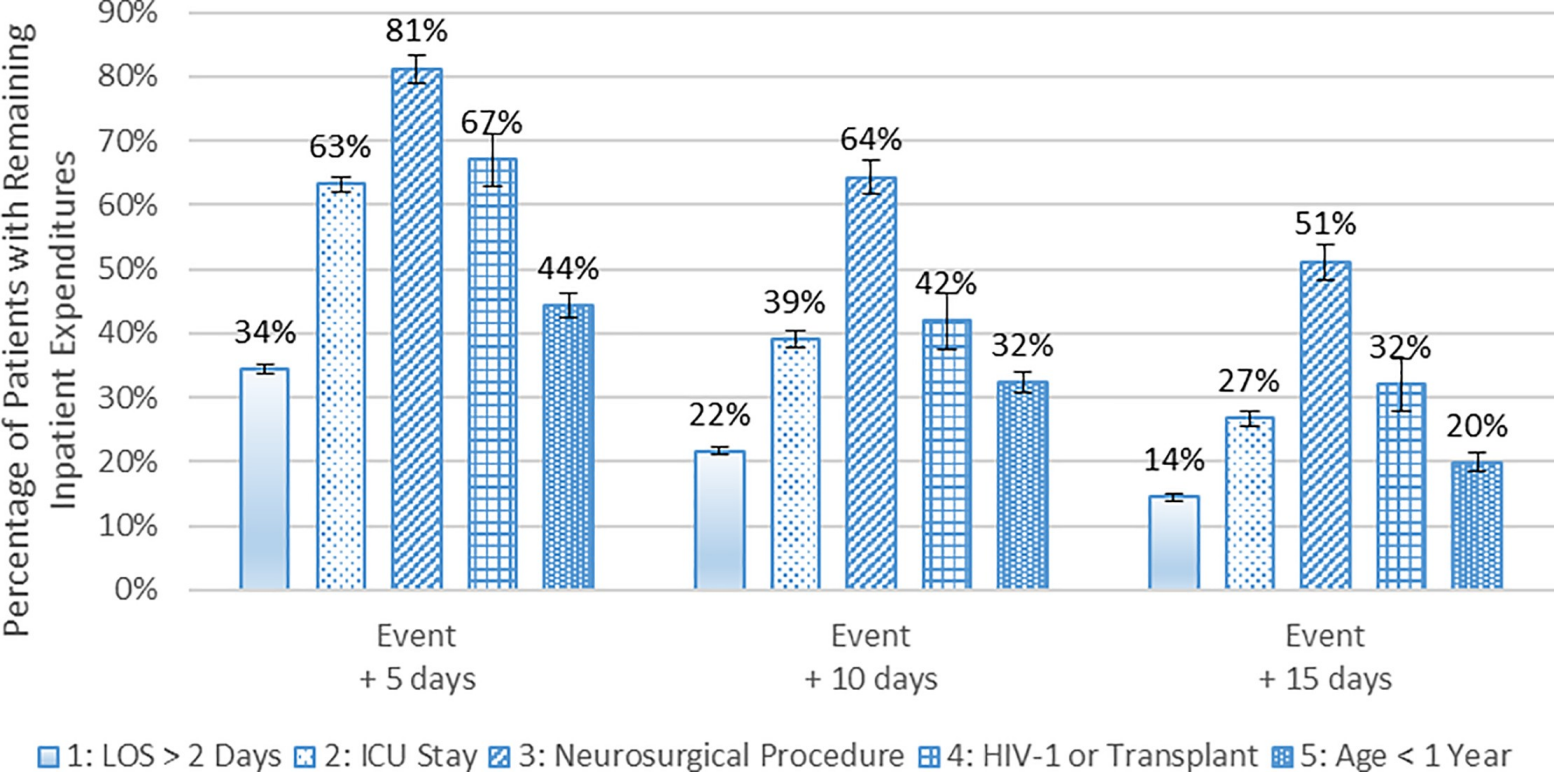
Percentage of hospitalized patients with meningitis who had inpatient expenditures after the subgroup-defining event, 2010 to 2014. Notes: The first 90 days and the last 90 days are excluded from the 2010 to 2014 period. For patients in Subgroup 4 (HIV-1 or Transplant) and Subgroup 5 (Age < 1 year), the subgroup-defining event day is the same as the initial hospital admission day because these patients could be classified into these subgroups upon admission. The values reported in the figure are the estimated percentages, and the error bars indicate the 95% confidence intervals of those estimates. Source: Authors’ analysis of 2010 to 2014 Truven Health MarketScan Commercial Claims and Encounters Database.

Fig 2 shows the percentage of patients hospitalized with meningitis who had remaining inpatient expenditures 5, 10 and 15 days after being classified into a patient subgroup (hereafter “subgroup-defining event”). For the subgroups of patients who had a neurosurgical procedure, HIV-1 infection or a previous organ transplant, or ICU stay, over half had inpatient expenditures for at least five days following the subgroup-defining event because these events usually occurred early in the patients’ episodes and these patients had longer LOS. Some events were known on the admission day, including whether a patient had HIV-1 infection or a previous organ transplant or was less than one year old. The other criteria were usually known early in an episode as well. For example, of 1,233 patients who had a neurosurgical procedure, 63% had the procedure on or before the third day of their episode. The percentages of these patients with inpatient expenditures remaining 5, 10 and 15 days after the procedure were 81%, 64% and 51%, respectively. Even more starkly, for the 5,894 patients with an ICU stay, 85% of these patients began an ICU stay on the first day of their episode. The percentages of these patients with inpatient expenditures remaining 5, 10 and 15 days after the initiation of an ICU stay were 63%, 39% and 27%, respectively.

**Fig 3:**
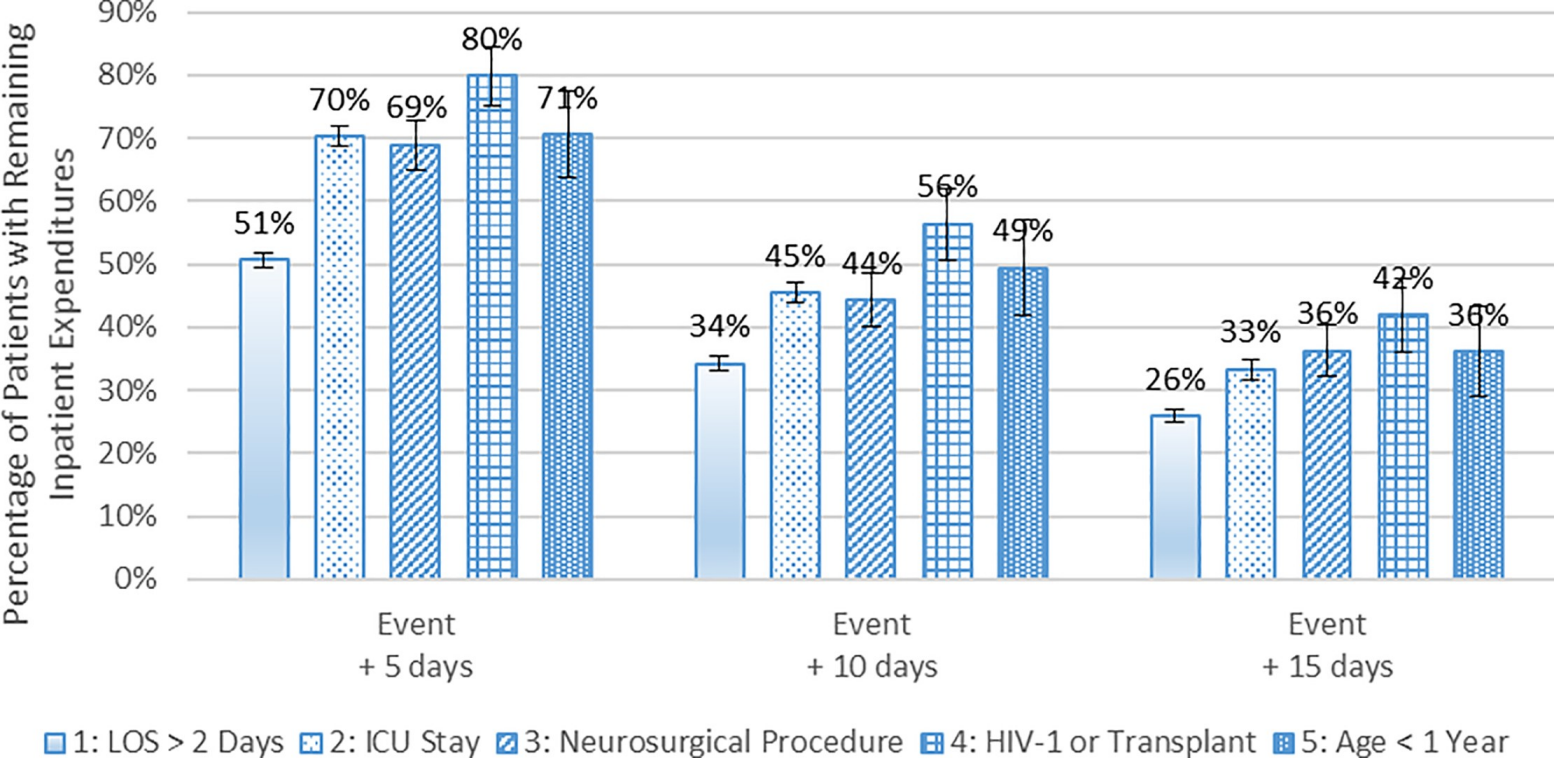
Percentage of patients with encephalitis who had inpatient expenditures after the subgroup-defining event, 2010 to 2014. Notes: The first 90 days and the last 90 days are excluded from the 2010 to 2014 period. For patients in Subgroup 4 (HIV-1 or Transplant) and Subgroup 5 (Age < 1 year), the subgroup-defining event day is the same as the initial hospital admission day because these patients could be classified into these subgroups upon admission. The values reported in the figure are the estimated percentages, and the error bars indicate the 95% confidence intervals of those estimates. Source: Authors’ analysis of 2010 to 2014 Truven Health MarketScan Commercial Claims and Encounters Database.

Fig 3 shows the percentage of patients hospitalized with encephalitis who had remaining inpatient expenditures 5, 10 and 15 days after the patient subgroup-defining event. For the subgroups of patients who had HIV-1 infection or a previous organ transplant, a neurosurgical procedure, or an ICU stay, over half had inpatient expenditures for at least five days following the subgroup-defining event because these events usually occurred early in the patients’ episodes and these patients had longer LOS. As stated above, some events were known on the admission day, including whether a patient had HIV-1 infection or a previous organ transplant or was less than one year old. For example, the percentages of 289 patients with HIV-1 infection or a previous organ transplant with inpatient expenditures remaining 5, 10 and 15 days after the initial hospital admission were 80%, 56% and 42%, respectively. The other criteria were usually known early in an episode as well. For example, of 535 patients who had a neurosurgical procedure, 53% had the procedure on or before the third day of their episode. The percentages of these patients with inpatient expenditures remaining 5, 10 and 15 days after the procedure were 69%, 44% and 36%, respectively. Furthermore, for the 3,430 patients with an ICU stay, 82% of these patients began an ICU stay on the first day of their episode. The percentages of these patients with inpatient expenditures remaining 5, 10 and 15 days after the initiation of an ICU stay were 70%, 45% and 33%, respectively.

**Fig 4:**
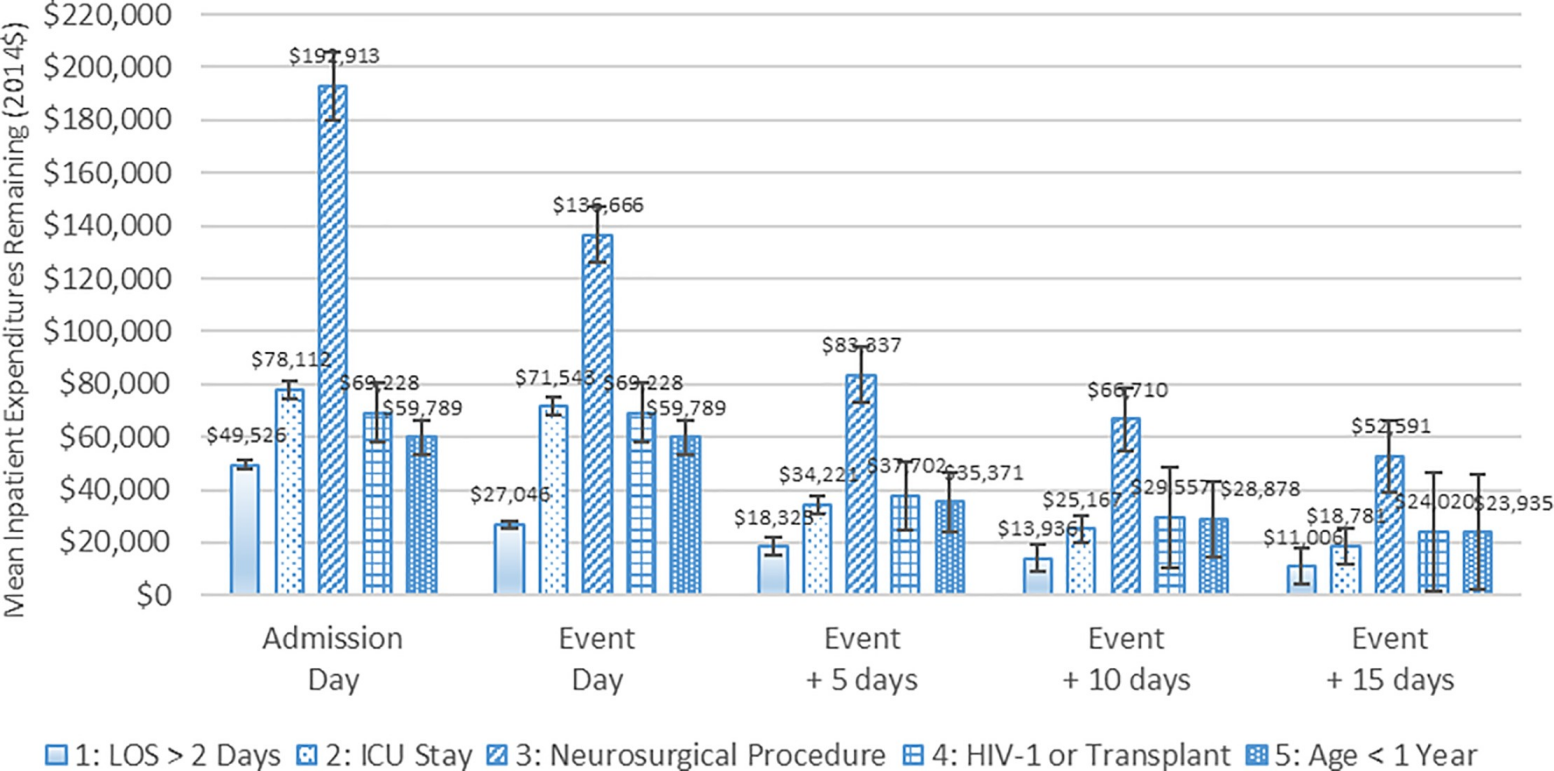
Mean inpatient healthcare expenditures remaining after the subgroup-defining event day for patients with meningitis, 2010 to 2014. Notes: The first 90 days and the last 90 days are excluded from the 2010 to 2014 period. For patients in Subgroup 4 (HIV-1 or Transplant) and Subgroup 5 (Age < 1 year), the subgroup-defining event day is the same as the initial hospital admission day because these patients could be classified into these subgroups upon admission. The values reported in the figure are the estimated means, and the error bars indicate the 95% confidence intervals of those estimates. Source: Authors’ analysis of 2010 to 2014 Truven Health MarketScan Commercial Claims and Encounters Database.

Fig 4 shows the mean inpatient healthcare expenditures remaining for patients with meningitis in each patient subgroup at the following points in time: at admission; on the subgroup-defining event day; and 5, 10 and 15 days after that event. The expenditures were averaged among all patients in a subgroup, including those whose episodes had ended before the above points in time. Each subgroup had significant remaining expenditures at 5 and 10 days after the event. For example, mean inpatient expenditures remaining by patient subgroup five days subsequent to the subgroup-defining event were as follows: neurosurgical procedure ($83,337), HIV-1 or a previous organ transplant ($37,702), less than one year old ($35,371), ICU stay ($34,221) and LOS greater than two days ($18,325). Turning to the subgroup of patients with highest expenditures after the subgroup-defining event—those who had a neurosurgical procedure—their mean expenditures totaled $192,913, of which, $83,337, $66,710 and $52,591 remained 5, 10 and 15 days after their neurosurgical procedure, respectively. Thus, had all 1,233 patients in this subgroup received an advanced diagnostic test on the day of their neurosurgical procedure, there would have been significant inpatient expenditures remaining—even 15 days after the test was administered. A similar case could be made for the patients in the other subgroups, even though their mean expenditures remaining 5, 10 and 15 days after their subgroup-defining event were generally not as high.

**Fig 5:**
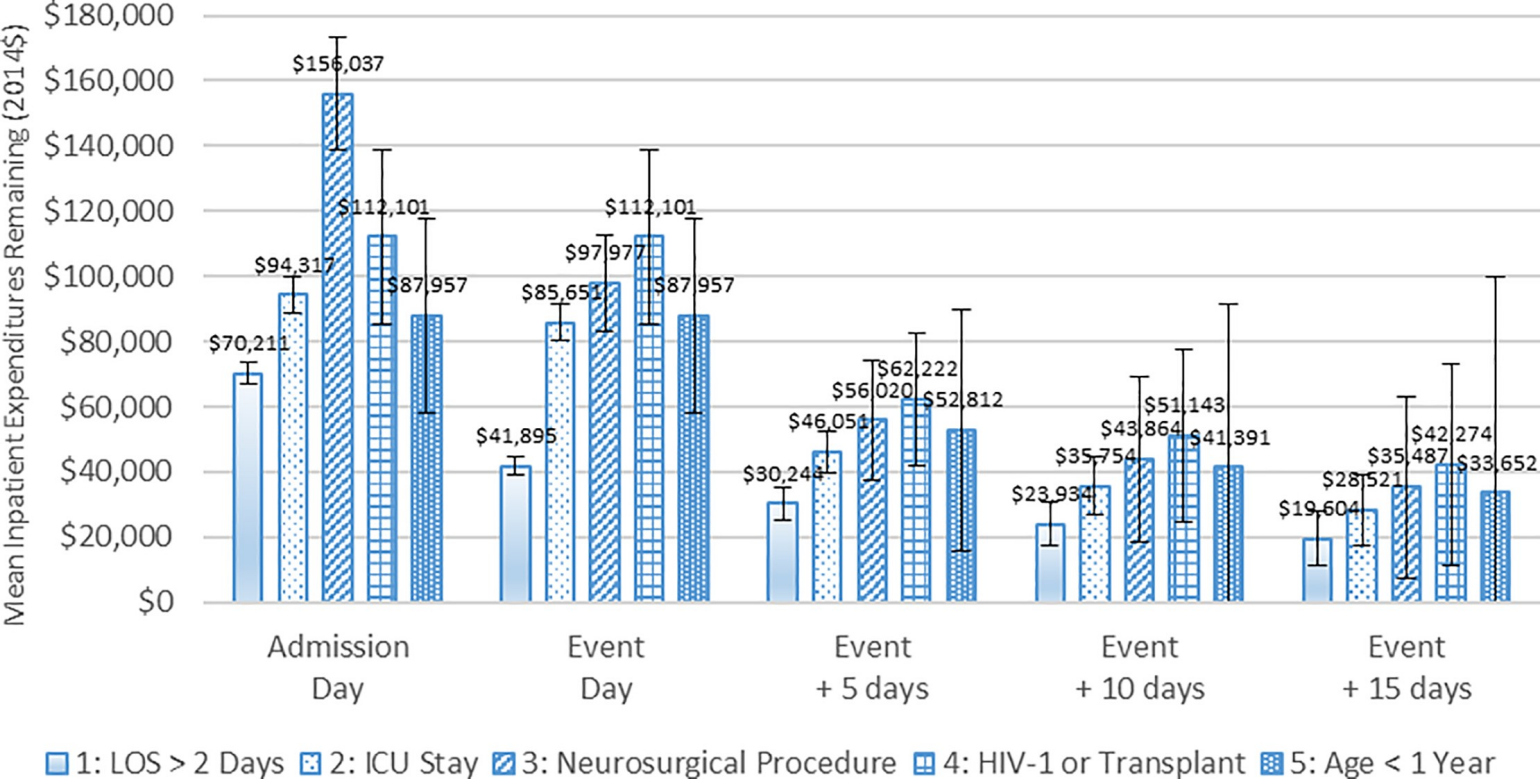
Mean inpatient healthcare expenditures remaining after the subgroup-defining event day for patients with encephalitis, 2010 to 2014. Notes: The first 90 days and the last 90 days are excluded from the 2010 to 2014 period. For patients in Subgroup 4 (HIV-1 or Transplant) and Subgroup 5 (Age < 1 year), the subgroup-defining event day is the same as the initial hospital admission day because these patients could be classified into these subgroups upon admission. The values reported in the figure are the estimated means, and the error bars indicate the 95% confidence intervals of those estimates. Source: Authors’ analysis of 2010 to 2014 Truven Health MarketScan Commercial Claims and Encounters Database.

Fig 5 is analogous to Fig 4, but includes patients with encephalitis. Each subgroup had significant remaining expenditures at five days after the event. For example, mean inpatient expenditures remaining by patient subgroup five days subsequent to the subgroup-defining event were as follows: HIV-1 or a previous organ transplant ($62,222), neurosurgical procedure ($56,020), less than one year old ($52,812), ICU stay ($46,051), and LOS greater than two days ($30,244). Turning to the subgroup of patients with highest expenditures after the subgroup-defining event—those who had HIV-1 infection or a previous organ transplant—their mean expenditures totaled $112,101, of which, $62,222, $51,143 and $42,274 remained 5, 10 and 15 days after their subgroup-defining event, respectively, which occurred for these patients on their admission day. Thus, had all 289 patients in this subgroup received an advanced diagnostic test on day of their hospital admission, there would have been significant inpatient expenditures remaining—even 15 days after the test was administered. Except for patients less than one year old, a similar case could be made for the patients in the other subgroups.

Table 4 shows the breakeven percentage reduction of the mean remaining healthcare expenditures five days after the sub-group defining event for patients hospitalized with meningitis or encephalitis. The breakeven percentage reduction is calculated for each of the five patient subgroups, which have different estimated mean expenditures remaining (as shown in the table), by varying the probability of an advanced diagnostic test changing care from 1% to 20% because the test will not affect all patients’ care. For example, for patients with meningitis who had a neurosurgical procedure, if the probability of the diagnostic test changing care was 10%, then a $2,000 test would break even if it reduced the mean remaining expenditures of $83,337 five days after the test by 24%. As the probability of the test changing care increases, then the percentage reduction in mean remaining expenditures to break even decreases: for these patients with meningitis, the breakeven percentage reduction decreases to 12% if the probability of changing care was 20%. For the patient subgroups 2–5, if the probability of changing care is at least 20%, then the breakeven percentage reduction for patients with meningitis is below 30% and for patients with encephalitis is below 25%.

**Table 4:** Percentage reduction in mean remaining healthcare expenditures 5 days after subgroup-defining event to break even on a $2,000 advanced diagnostic test for patients hospitalized with meningitis or encephalitis by probability of the test changing care and patient sub-group.

Patients Hospitalized with Meningitis					
Patient Subgroup	1: LOS > 2 Days	2: ICU Stay	3: Neurosurgical Procedure	4: HIV-1 or Transplant	5: Age < 1 Year
Remaining HCE (mean)	$18,325	$34,221	$83,337	$37,702	$35,371
Probability of Change Care	Percentage Reduction in Mean Remaining HCE Needed to Break Even				
1%	>100%	>100%	>100%	>100%	>100%
5%	>100%	>100%	48%	>100%	>100%
10%	>100%	58%	24%	53%	57%
15%	73%	39%	16%	35%	38%
20%	55%	29%	12%	27%	28%
Patients Hospitalized with Encephalitis					
Patient Subgroup	1: LOS > 2 Days	2: ICU Stay	3: Neurosurgical Procedure	4: HIV-1 or Transplant	5: Age < 1 Year
Remaining HCE (mean)	$30,244	$46,051	$56,020	$62,222	$52,812
Probability of Change Care	Percentage Reduction in Mean Remaining HCE Needed to Break Even				
1%	>100%	>100%	>100%	>100%	>100%
5%	>100%	87%	71%	64%	76%
10%	66%	43%	36%	32%	38%
15%	44%	29%	24%	21%	25%
20%	33%	22%	18%	16%	19%

Notes: HCE: healthcare expenditures; Remaining HCE is the mean healthcare expenditures remaining 5 days after the subgroup-defining event.

Source: Authors’ analysis of 2010 to 2014 Truven Health MarketScan Commercial Claims and Encounters Database

## Discussion

This study found that patients hospitalized with meningitis or encephalitis had substantial inpatient healthcare expenditures that varied widely. Mean inpatient expenditures were $36,891 (SD = $92,636) and $60,181 (SD = $130,276) for patients with meningitis or encephalitis, respectively. These amounts were significantly higher than the average, as mean inpatient expenditures (excluding inpatient physician services) across hospital admissions for all conditions were estimated to be only $21,433 [29]. Our results were based on actual amounts paid, which is the appropriate measure to use when estimating expenditures that could be impacted by an advanced diagnostic test, as amounts paid are usually significantly lower than charges [18, 19]. Our mean LOS results per hospitalization (not per episode, which may include two or more hospitalizations) for patients with meningitis or encephalitis were 7.6 days and 10.6 days, respectively, or approximately 1–2 days lower than previous estimates of 9.1 days [1] and 12.6 days [4], respectively. These differences are likely because those studies also included patients 65+ years old.

Broad-spectrum infectious disease diagnostic tests (e.g., mNGS) can modify a patient’s treatment through several channels: 1) identifying unusual and/or unexpected, treatable infections leading to more rapidly tailored therapy [11–14, 30]; 2) identifying infections with a poor prognosis [31–33], which could lead to cessation of aggressive therapeutic and diagnostic maneuvers; and 3) accelerating the initiation of immunosuppressive therapy for patients in whom an autoimmune cause of encephalitis is being considered. However, we recognize that whether an infection is identified, there are other critical factors that determine whether an advanced diagnostic test impacts a particular patient’s clinical outcome and healthcare-expenditures including sample-to-answer turnaround time, whether their treatment is modified based on the test results, and the impact of the treatment modification.

Here, we showed that patient subgroups with higher healthcare utilization and expenditures could be identified early in their hospitalization, based on the following clinical, demographic and healthcare utilization criteria gleaned from the medical literature: a neurosurgical procedure, an ICU stay, HIV-1 infection or a previous organ transplant, less than one year old, or a hospital LOS greater than two days [3, 4, 25–27]. When designing future cost-effectiveness trials for advanced diagnostic tests, these selected patient subgroups may serve as high-value targets for assessing a test’s impact on the course of care, including its effectiveness on improving patient health and reducing LOS, healthcare utilization, and healthcare expenditures [34]. Moreover, these findings can help guide and appropriately power future interventional diagnostic and treatment trials for these highly morbid syndromes.

This study has the following limitations. First, the study did not attempt to use more sophisticated modeling techniques to predict which patients were most at risk for long LOS and high inpatient healthcare expenditures because of the sophisticated statistical methods that would need to be described and presented, such as k-fold, cross-validation training and test datasets with many additional variables (e.g., Elixhauser Comorbidity Index). The purpose of our approach was to demonstrate the feasibility of grouping patients *a priori* using widely recognized, high-risk criteria to predict which patient subgroups were likely to have long LOS and high inpatient healthcare expenditures [3, 4, 25–27]. Second, while we recognize that healthcare expenditures will vary based on the underlying etiology of a patient’s meningitis or encephalitis, we did not perform etiology-specific healthcare-expenditure analyses because billing codes (without clinical adjudication) provide a flawed and incomplete picture of meningitis and encephalitis etiologies. Related, we are not aware of a study that has estimated the sensitivity and specificity of using ICM-9-CM diagnosis codes, but they were the best measure within the data. Third, the Truven dataset does not provide additional detail on the degree that patients with HIV-1 infection are immunocompromised. Fourth, it was not possible to separate inpatient expenditures to diagnose and treat meningitis or encephalitis versus a co-morbid condition. Even if this distinction were possible, we would be reluctant to separate expenditures because meningitis and encephalitis can lead to co-morbid conditions (e.g., a fall, multi-organ failure, and respiratory compromise requiring ventilation). Fifth, due to contractual provisions between insurers and healthcare providers regarding reimbursement, we were not always able to allocate expenditures by type of healthcare utilization, such as separating ICU expenditures from general floor hospital expenditures for some episodes (Table 2) and separating inpatient physician expenditures from hospital expenditures, as these expenditures may have been bundled. However, this limitation did not significantly affect our ability to estimate total inpatient expenditures, a critical estimate of our study. Sixth, we allocated general floor hospital facility expenditures evenly across a hospital stay, which is appropriate for insurers that reimburse hospitals on a per diem basis. For insurers that reimburse hospitals on a case-rate basis (e.g., diagnosis related groups), these expenditures are fully incurred upon admission; however, outlier payments that apply to patients who have exceptionally long LOS are not incurred until after the patient is discharged. In the long run, case rates are adjusted to incorporate changes in hospitalization costs, including reduced LOS from advanced diagnostic tests.

Future research is needed on the validation of additional clinical, demographic and healthcare utilization criteria for identifying which subgroups of patients would benefit most from an advanced diagnostic test (e.g. mNGS), including the incorporation of data from prospective clinical trials and methods from machine learning-based approaches for prediction. That research is critical in the evaluation of advanced diagnostic tests and their potential benefit to patients and healthcare payers.

## Supporting information

S1 Appendix (Table A) ICD-9-CM Codes and Descriptions Used to Identify Patients with Meningitis. (Table B) ICD-9-CM Codes and Descriptions Used to Identify Patients with Encephalitis. (Table C) Neurosurgical Procedure CPT Codes for Patients with Meningitis. (Table D) Neurosurgical Procedure CPT Codes for Patients with Encephalitis. (Table E) Number of Patients by Patient Subgroup Combination.
(DOCX)
