# Supplementary material for: Exploratory analysis of the potential for advanced diagnostic testing to reduce healthcare expenditures of patients hospitalized with meningitis or encephalitis
Source: PLoS One. 2020 Jan 15;15(1):e0226895. doi: 10.1371/journal.pone.0226895 (PMC6961903; doi:10.1371/journal.pone.0226895)
Supplement: S1 Appendix — (Table A) ICD-9-CM Codes and Descriptions Used to Identify Patients with Meningitis. (Table B) ICD-9-CM Codes and Descriptions Used to Identify Patients with Encephalitis. (Table C) Neurosurgical Procedure CPT Codes for Patients with Meningitis. (Table D) Neurosurgical Procedure CPT Codes for Patients with Encephalitis. (Table E) Number of Patients by Patient Subgroup Combination. (DOCX) [file pone.0226895.s001.docx]

Title: Exploratory analysis of the potential for advanced diagnostic testing to reduce healthcare expenditures of patients hospitalized with meningitis or encephalitis

# S1 Appendix

The supporting information includes the following five tables referenced in the main study.

Table A lists the ICD-9-CM codes we used to identify patients with meningitis, based on the codes used in previous studies.^1-3^ If the code was listed in at least one study, we included it.

Table A: ICD-9-CM Codes and Descriptions Used to Identify Patients with Meningitis

| ICD-9-CM Code | Description |
| --- | --- |
| 003.21 | Salmonella meningitis |
| 013.0 | Tuberculous meningitis unspecified examination |
| 013.01 | Tuberculous meningitis bacteriological or histological examination not done |
| 013.02 | Tuberculous meningitis bacteriological or histological examination results unknown (at present) |
| 013.03 | Tuberculous meningitis tubercle bacilli found (in sputum) by microscopy |
| 013.04 | Tuberculous meningitis tubercle bacilli not found (in sputum) by microscopy but found by bacterial culture |
| 013.05 | Tuberculous meningitis tubercle bacilli not found by bacteriological examination but tuberculosis confirmed histologically |
| 013.06 | Tuberculous meningitis tubercle bacilli not found by bacteriological or histological examination but tuberculosis confirmed by other methods (inoculation of animals) |
| 036.0 | Meningococcal meningitis |
| 047.0 | Meningitis due to coxsachkie virus |
| 047.1 | Meningitis due to echo virus |
| 047.8 | Other specified viral meningitis |
| 047.9 | Unspecified viral meningitis |
| 049.0 | Meningitis due to lymphocytic choriomeningitis virus |
| 049.1 | Non-arthopod borne meningitis due to adenovirus |
| 053.0 | Herpes zoster with meningitis |
| 054.72 | Herpes simplex meningitis |
| 072.1 | Mumps meningitis |
| 090.42 | Congenital syphilitic meningitis |
| 091.81 | Acute syphilitic meningitis (secondary) |
| 094.2 | Syphilitic meningitis |
| 098.82 | Gonococcal meningitis |
| 100.81 | Leptospiral meningitis (aseptic) |
| 112.83 | Candidal meningitis |
| 114.2 | Coccidioidal meningitis |
| 115.01 | Histoplasma capsulatum meningitis |
| 115.11 | Histoplasma duboisii meningitis |
| 115.91 | Histoplasmosis meningitis unspecified |
| 320.0 | Hemophilus meningitis |
| 320.1 | Pneumococcal meningitis |
| 320.2 | Streptococcal meningitis |
| 320.3 | Staphylococcal meningitis |
| 320.7 | Meningitis in other bacterial diseases classified elsewhere |
| 320.81 | Anaerobic meningitis |
| 320.82 | Meningitis due to gram-negative bacteria not elsewhere classified |
| 320.89 | Meningitis due to other specified bacteria |
| 320.9 | Meningitis due to unspecified bacterium |
| 321.0 | Cryptococcal meningitis |
| 321.1 | Meningitis in other fungal diseases |
| 321.2 | Meningitis due to viruses not elsewhere classified |
| 321.3 | Meningitis due to trypanosomiasis |
| 321.4 | Meningitis in sarcoidosis |
| 321.8 | Meningitis due to other nonbacterial organisms classified |
| 322.0 | Nonpyogenic Meningitis |
| 322.1 | Eosinophilic Meningitis |
| 322.2 | Chronic Meningitis |
| 322.9 | Meningitis Unspecified |

Sources: Nigrovic et al. (2013),^1^ Takhar et al. (2012)^2^ and Holmquist (2008)^3^

Table B lists ICD-9-CM codes we used to identify patients with encephalitis, based on the codes used in a previous study.^4^

Table B: ICD-9-CM Codes and Descriptions Used to Identify Patients with Encephalitis

| ICD-9-CM Code (1) | Previous Code  (if applicable) | Description |
| --- | --- | --- |
| Viral |  |  |
| 045.0 |  | Acute bulbar poliomyelitis |
| 049.8 |  | Other specified non-arthopodborne viral encephalitis |
| 058.21, 058.29 (2007) | 054.3 | Herpetic meningoencephalitis |
| 056.01 |  | Encephalitis due to rubella |
| 062.0 |  | Japanese encephalitis |
| 062.1 |  | Western equine encephalitis |
| 062.2 |  | Eastern equine encephalitis |
| 062.3 |  | St. Louis encephalitis |
| 062.4 |  | Australian encephalitis |
| 062.5 |  | California virus encephalitis |
| 062.8 |  | Other specified mosquitoborne viral encephalitis |
| 063.0 |  | Russian spring-summer encephalitis |
| 063.1 |  | Louping ill |
| 063.2 |  | Central European encephalitis |
| 063.8 |  | Other specified tickborne encephalitis |
| 066.2 |  | Venezuelan equine encephalitis |
| 066.41 |  | West Nile fever with encephalitis |
| 071 |  | Rabies |
| 072.2 |  | Mumps encephalitis |
| 323.01 (2006) | 323 | Encephalitis in viral diseases classified elsewhere |
| Other Infections |  |  |
| 013.6 |  | Tuberculous encephalitis |
| 036.1 |  | Meningococcal encephalitis |
| 090.41 |  | Congenital syphilitic encephalitis |
| 094.81 |  | Syphilitic encephalitis |
| 130.0 |  | Toxoplasma encephalitis |
| 136.29 (2008) | 136.2 | Meningoencephalitis due to Naegleria |
| 323.1 |  | Encephalitis in rickettsial diseases classified elsewhere |
| 323.2 |  | Encephalitis in protozoal diseases classified elsewhere |
| 323.41 (2006) | 323.4 | Other encephalitis due to infection classified elsewhere |
| Postimmunization |  |  |
| 323.51 (2006) | 323.5 | Encephalitis following immunization procedures |
| Postinfectious |  |  |
| 052.0 |  | Postvaricella encephalitis |
| 055.0 |  | Postmeasles encephalitis |
| 323.61, 323.62 (2006) | 323.6 | Postinfectious encephalitis (including infectious acute disseminated encephalomyelitis) |
| Toxic |  |  |
| 323.71 (2006) | 323.7 | Toxic encephalitis |
| Other Specified |  |  |
| 323.81 (2006) | 323.8 | Other causes of encephalitis (including noninfectious acute disseminated encephalomyelitis) |
| Unspecified Etiology |  |  |
| 323.9 |  | Unspecified cause of encephalitis |
| 049.9 |  | Unspecified viral encephalitis |
| 062.9 |  | Unspecified mosquitoborne encephalitis |
| 063.9 |  | Unspecified tickborne viral encephalitis |
| 064 |  | Viral encephalitis transmitted by other and unspecified arthropods |

1. Includes year code began, if revised.

Source: Vora et al. (2014)^4^

Table C lists the CPT codes we used to identify the 1,233 patients with meningitis who had a neurosurgical procedure, most commonly related to neuromonitoring and shunting. Some of these patients had more than one neurosurgical procedure so the table includes the counts of each patient’s first procedure as well as the counts of all their neurosurgical procedures. The codes were selected based on analyzing the Truven data and authors’ expertise, but the patient count for some codes was zero.

Table C: Neurosurgical Procedure CPT Codes for Patients with Meningitis

| No. | CPT Code | Description | First Procedure Count  N=1,233 | All Procedures Count  N=4,315 | Cumulative Percent (first procedure) |
| --- | --- | --- | --- | --- | --- |
| 1 | 61210 | Pierce skull implant device | 186 | 418 | 15% |
| 2 | 61107 | Drill skull for implantation | 162 | 346 | 28% |
| 3 | 61070 | Brain canal shunt procedure | 92 | 693 | 36% |
| 4 | 61624 | Transcath occlusion cns | 55 | 151 | 40% |
| 5 | 61320 | Open skull for drainage | 48 | 83 | 44% |
| 6 | 61020 | Remove brain cavity fluid | 46 | 197 | 48% |
| 7 | 61781 | Scan proc cranial intra | 45 | 348 | 51% |
| 8 | 61343 | Incise skull (press relief) | 39 | 70 | 55% |
| 9 | 61618 | Repair dura | 29 | 137 | 57% |
| 10 | 61782 | Scan proc cranial extra | 29 | 85 | 59% |
| 11 | 61215 | Insert brain-fluid device | 27 | 80 | 61% |
| 12 | 61312 | Open skull for drainage | 26 | 70 | 64% |
| 13 | 61510 | Removal of brain lesion | 25 | 61 | 66% |
| 14 | 61512 | Remove brain lining lesion | 25 | 37 | 68% |
| 15 | 61697 | Brain aneurysm repr complx | 23 | 77 | 70% |
| 16 | 61304 | Open skull for exploration | 22 | 47 | 71% |
| 17 | 61548 | Removal of pituitary gland | 16 | 38 | 73% |
| 18 | 61154 | Pierce skull & remove clot | 15 | 33 | 74% |
| 19 | 61514 | Removal of brain abscess | 15 | 38 | 75% |
| 20 | 61140 | Pierce skull for biopsy | 12 | 19 | 76% |
| 21 | 61751 | Brain biopsy w/ct/mr guide | 12 | 21 | 77% |
| 22 | 61050 | Remove brain canal fluid | 11 | 12 | 78% |
| 23 | 61518 | Removal of brain lesion | 10 | 44 | 79% |
| 24 | 61795 | Brain surgery using computer | 10 | 80 | 79% |
| 25 | 61583 | Craniofacial approach skull | 9 | 26 | 80% |
| 26 | 61000 | Remove cranial cavity fluid | 8 | 8 | 81% |
| 27 | 61150 | Pierce skull for drainage | 8 | 14 | 82% |
| 28 | 61533 | Implant brain electrodes | 8 | 11 | 82% |
| 29 | 61619 | Repair dura | 8 | 47 | 83% |
| 30 | 61700 | Brain aneurysm repr simple | 8 | 16 | 83% |
| 31 | 61750 | Incise skull/brain biopsy | 8 | 14 | 84% |
| 32 | 61120 | Burr hole for puncture | 7 | 26 | 85% |
| 33 | 61313 | Open skull for drainage | 7 | 42 | 85% |
| 34 | 61322 | Decompressive craniotomy | 7 | 77 | 86% |
| 35 | 61520 | Removal of brain lesion | 7 | 23 | 86% |
| 36 | 61026 | Injection into brain canal | 6 | 121 | 87% |
| 37 | 61105 | Twist drill hole | 6 | 6 | 87% |
| 38 | 61580 | Craniofacial approach skull | 6 | 25 | 88% |
| 39 | 61584 | Orbitocranial approach/skull | 6 | 21 | 88% |
| 40 | 61591 | Infratemporal approach/skull | 6 | 20 | 89% |
| 41 | 61595 | Transtemporal approach/skull | 6 | 19 | 89% |
| 42 | 61590 | Infratemporal approach/skull | 5 | 12 | 90% |
| 43 | 61867 | Implant neuroelectrode | 5 | 18 | 90% |
| 44 | 61108 | Drill skull for drainage | 4 | 10 | 90% |
| 45 | 61305 | Open skull for exploration | 4 | 9 | 91% |
| 46 | 61321 | Open skull for drainage | 4 | 13 | 91% |
| 47 | 61501 | Remove infected skull bone | 4 | 7 | 91% |
| 48 | 61526 | Removal of brain lesion | 4 | 9 | 92% |
| 49 | 61538 | Removal of brain tissue | 4 | 8 | 92% |
| 50 | 61542 | Removal of brain tissue | 4 | 4 | 92% |
| 51 | 61592 | Orbitocranial approach/skull | 4 | 12 | 93% |
| 52 | 61601 | Resect/excise cranial lesion | 4 | 61 | 93% |
| 53 | 61783 | Scan proc spinal | 4 | 6 | 93% |
| 54 | 61055 | Injection into brain canal | 3 | 3 | 94% |
| 55 | 61250 | Pierce skull & explore | 3 | 5 | 94% |
| 56 | 61315 | Open skull for drainage | 3 | 15 | 94% |
| 57 | 61516 | Removal of brain lesion | 3 | 8 | 94% |
| 58 | 61521 | Removal of brain lesion | 3 | 5 | 95% |
| 59 | 61543 | Removal of brain tissue | 3 | 5 | 95% |
| 60 | 61544 | Remove & treat brain lesion | 3 | 10 | 95% |
| 61 | 61545 | Excision of brain tumor | 3 | 11 | 95% |
| 62 | 61616 | Resect/excise lesion skull | 3 | 34 | 96% |
| 63 | 61686 | Intracranial vessel surgery | 3 | 9 | 96% |
| 64 | 61323 | Decompressive lobectomy | 2 | 19 | 96% |
| 65 | 61345 | Relieve cranial pressure | 2 | 10 | 96% |
| 66 | 61458 | Incise skull for brain wound | 2 | 7 | 96% |
| 67 | 61519 | Remove brain lining lesion | 2 | 4 | 96% |
| 68 | 61524 | Removal of brain lesion | 2 | 15 | 97% |
| 69 | 61537 | Removal of brain tissue | 2 | 7 | 97% |
| 70 | 61559 | Excision of skull/sutures | 2 | 2 | 97% |
| 71 | 61582 | Craniofacial approach skull | 2 | 8 | 97% |
| 72 | 61586 | Resect nasopharynx skull | 2 | 4 | 97% |
| 73 | 61597 | Transcondylar approach/skull | 2 | 4 | 97% |
| 74 | 61598 | Transpetrosal approach/skull | 2 | 9 | 98% |
| 75 | 61605 | Resect/excise cranial lesion | 2 | 15 | 98% |
| 76 | 61626 | Transcath occlusion non-cns | 2 | 8 | 98% |
| 77 | 61760 | Implant brain electrodes | 2 | 3 | 98% |
| 78 | 61253 | Pierce skull & explore | 1 | 1 | 98% |
| 79 | 61316 | Implt cran bone flap to abdo | 1 | 7 | 98% |
| 80 | 61332 | Explore/biopsy eye socket | 1 | 1 | 98% |
| 81 | 61460 | Incise skull for surgery | 1 | 1 | 98% |
| 82 | 61522 | Removal of brain abscess | 1 | 6 | 98% |
| 83 | 61530 | Removal of brain lesion | 1 | 4 | 99% |
| 84 | 61536 | Removal of brain lesion | 1 | 2 | 99% |
| 85 | 61539 | Removal of brain tissue | 1 | 1 | 99% |
| 86 | 61541 | Incision of brain tissue | 1 | 1 | 99% |
| 87 | 61546 | Removal of pituitary gland | 1 | 1 | 99% |
| 88 | 61566 | Removal of brain tissue | 1 | 1 | 99% |
| 89 | 61567 | Incision of brain tissue | 1 | 2 | 99% |
| 90 | 61571 | Incise skull for brain wound | 1 | 1 | 99% |
| 91 | 61581 | Craniofacial approach skull | 1 | 1 | 99% |
| 92 | 61585 | Orbitocranial approach/skull | 1 | 2 | 99% |
| 93 | 61596 | Transcochlear approach/skull | 1 | 2 | 99% |
| 94 | 61600 | Resect/excise cranial lesion | 1 | 18 | 99% |
| 95 | 61608 | Resect/excise cranial lesion | 1 | 18 | 100% |
| 96 | 61623 | Endovasc tempory vessel occl | 1 | 3 | 100% |
| 97 | 61682 | Intracranial vessel surgery | 1 | 6 | 100% |
| 98 | 61698 | Brain aneurysm repr complx | 1 | 11 | 100% |
| 99 | 61860 | Implant neuroelectrodes | 1 | 4 | 100% |
| 100 | 61863 | Implant neuroelectrode | 1 | 1 | 100% |
| 101 | 61880 | Revise/remove neuroelectrode | 1 | 1 | 100% |
| 102 | 61001 | Remove cranial cavity fluid | 0 | 8 | 100% |
| 103 | 61151 | Pierce skull for drainage | 0 | 1 | 100% |
| 104 | 61156 | Pierce skull for drainage | 0 | 2 | 100% |
| 105 | 61314 | Open skull for drainage | 0 | 4 | 100% |
| 106 | 61330 | Decompress eye socket | 0 | 0 | 100% |
| 107 | 61333 | Explore orbit/remove lesion | 0 | 2 | 100% |
| 108 | 61340 | Subtemporal decompression | 0 | 0 | 100% |
| 109 | 61440 | Incise skull for surgery | 0 | 0 | 100% |
| 110 | 61490 | Incise skull for surgery | 0 | 0 | 100% |
| 111 | 61500 | Removal of skull lesion | 0 | 0 | 100% |
| 112 | 61517 | Implt brain chemotx add-on | 0 | 3 | 100% |
| 113 | 61531 | Implant brain electrodes | 0 | 0 | 100% |
| 114 | 61534 | Removal of brain lesion | 0 | 2 | 100% |
| 115 | 61535 | Remove brain electrodes | 0 | 5 | 100% |
| 116 | 61540 | Removal of brain tissue | 0 | 0 | 100% |
| 117 | 61556 | Incise skull/sutures | 0 | 0 | 100% |
| 118 | 61557 | Incise skull/sutures | 0 | 0 | 100% |
| 119 | 61558 | Excision of skull/sutures | 0 | 1 | 100% |
| 120 | 61570 | Remove foreign body brain | 0 | 4 | 100% |
| 121 | 61575 | Skull base/brainstem surgery | 0 | 0 | 100% |
| 122 | 61606 | Resect/excise cranial lesion | 0 | 13 | 100% |
| 123 | 61607 | Resect/excise cranial lesion | 0 | 1 | 100% |
| 124 | 61615 | Resect/excise lesion skull | 0 | 3 | 100% |
| 125 | 61630 | Intracranial angioplasty | 0 | 0 | 100% |
| 126 | 61635 | Intracran angioplsty w/stent | 0 | 0 | 100% |
| 127 | 61640 | Dilate ic vasospasm init | 0 | 26 | 100% |
| 128 | 61641 | Dilate ic vasospasm add-on | 0 | 21 | 100% |
| 129 | 61642 | Dilate ic vasospasm add-on | 0 | 9 | 100% |
| 130 | 61680 | Intracranial vessel surgery | 0 | 3 | 100% |
| 131 | 61684 | Intracranial vessel surgery | 0 | 2 | 100% |
| 132 | 61692 | Intracranial vessel surgery | 0 | 0 | 100% |
| 133 | 61702 | Inner skull vessel surgery | 0 | 0 | 100% |
| 134 | 61708 | Revise circulation to head | 0 | 0 | 100% |
| 135 | 61710 | Revise circulation to head | 0 | 1 | 100% |
| 136 | 61711 | Fusion of skull arteries | 0 | 2 | 100% |
| 137 | 61770 | Incise skull for treatment | 0 | 1 | 100% |
| 138 | 61796 | Srs cranial lesion simple | 0 | 0 | 100% |
| 139 | 61800 | Apply srs headframe add-on | 0 | 2 | 100% |
| 140 | 61868 | Implant neuroelectrde addl | 0 | 7 | 100% |
| 141 | 61885 | Insrt/redo neurostim 1 array | 0 | 1 | 100% |
| 142 | 61886 | Implant neurostim arrays | 0 | 4 | 100% |
| 143 | 61888 | Revise/remove neuroreceiver | 0 | 1 | 100% |
| 144 | 62010 | Treatment of head injury | 0 | 0 | 100% |
| 145 | 62100 | Repair brain fluid leakage | 0 | 0 | 100% |
| 146 | 62115 | Reduction of skull defect | 0 | 0 | 100% |
| 147 | 62116 | Reduction of skull defect | 0 | 0 | 100% |
| 148 | 62120 | Repair skull cavity lesion | 0 | 0 | 100% |
| 149 | 62121 | Incise skull repair | 0 | 0 | 100% |
| 150 | 62140 | Repair of skull defect | 0 | 0 | 100% |
| 151 | 62141 | Repair of skull defect | 0 | 0 | 100% |
| 152 | 62142 | Remove skull plate/flap | 0 | 0 | 100% |
| 153 | 62143 | Replace skull plate/flap | 0 | 0 | 100% |
| 154 | 62145 | Repair of skull & brain | 0 | 0 | 100% |
| 155 | 62146 | Repair of skull with graft | 0 | 0 | 100% |
| 156 | 62147 | Repair of skull with graft | 0 | 0 | 100% |
| 157 | 62148 | Retr bone flap to fix skull | 0 | 0 | 100% |
| 158 | 62160 | Neuroendoscopy add-on | 0 | 0 | 100% |
| 159 | 62161 | Dissect brain w/scope | 0 | 0 | 100% |
| 160 | 62162 | Remove colloid cyst w/scope | 0 | 0 | 100% |
| 161 | 62163 | Zneuroendoscopy w/fb removal | 0 | 0 | 100% |
| 162 | 62164 | Remove brain tumor w/scope | 0 | 0 | 100% |
| 163 | 62165 | Remove pituit tumor w/scope | 0 | 0 | 100% |
| 164 | 62180 | Establish brain cavity shunt | 0 | 0 | 100% |
| 165 | 62190 | Establish brain cavity shunt | 0 | 0 | 100% |
| 166 | 62192 | Establish brain cavity shunt | 0 | 0 | 100% |
| 167 | 62194 | Replace/irrigate catheter | 0 | 0 | 100% |
| 168 | 62200 | Establish brain cavity shunt | 0 | 0 | 100% |
| 169 | 62201 | Brain cavity shunt w/scope | 0 | 0 | 100% |
| 170 | 62220 | Establish brain cavity shunt | 0 | 0 | 100% |
| 171 | 62223 | Establish brain cavity shunt | 0 | 0 | 100% |
| 172 | 62225 | Replace/irrigate catheter | 0 | 0 | 100% |
| 173 | 62230 | Replace/revise brain shunt | 0 | 0 | 100% |
| 174 | 62252 | Csf shunt reprogram | 0 | 0 | 100% |
| 175 | 62256 | Remove brain cavity shunt | 0 | 0 | 100% |
| 176 | 62258 | Replace brain cavity shunt | 0 | 0 | 100% |
| 177 | 62267 | Interdiscal perq aspir dx | 0 | 0 | 100% |
| 178 | 62272 | Drain cerebro spinal fluid | 0 | 0 | 100% |

Source: Authors’ Analysis of 2010 to 2014 Truven Health MarketScan Commercial Claims and Encounters Database

Table D lists the CPT codes we used to identify the 535 patients with encephalitis who had a neurosurgical procedure, most commonly related to neuromonitoring and biopsy. Some of these patients had more than one neurosurgical procedure so the table includes the counts of each patient’s first procedure as well as the counts of all their neurosurgical procedures. The codes were selected based on analyzing the Truven data and authors’ expertise, but the patient count for some codes was zero.

Table D: Neurosurgical Procedure CPT Codes for Patients with Encephalitis

| No. | CPT Code | Description | First Procedure Count  N=535 | All Procedures Count  N=1,314 | Cumulative Percent (first procedure) |
| --- | --- | --- | --- | --- | --- |
| 1 | 61510 | Removal of brain lesion | 66 | 111 | 12% |
| 2 | 61107 | Drill skull for implantation | 46 | 76 | 21% |
| 3 | 61320 | Open skull for drainage | 45 | 81 | 29% |
| 4 | 61751 | Brain biopsy w/ct/mr guide | 44 | 69 | 38% |
| 5 | 61750 | Incise skull/brain biopsy | 41 | 58 | 45% |
| 6 | 61304 | Open skull for exploration | 34 | 52 | 52% |
| 7 | 61210 | Pierce skull implant device | 32 | 66 | 58% |
| 8 | 61514 | Removal of brain abscess | 27 | 46 | 63% |
| 9 | 61140 | Pierce skull for biopsy | 26 | 43 | 67% |
| 10 | 61070 | Brain canal shunt procedure | 12 | 60 | 70% |
| 11 | 61782 | Scan proc cranial extra | 11 | 47 | 72% |
| 12 | 61150 | Pierce skull for drainage | 10 | 20 | 74% |
| 13 | 61020 | Remove brain cavity fluid | 7 | 10 | 75% |
| 14 | 61312 | Open skull for drainage | 7 | 17 | 76% |
| 15 | 61512 | Remove brain lining lesion | 6 | 11 | 77% |
| 16 | 61624 | Transcath occlusion cns | 6 | 24 | 79% |
| 17 | 61518 | Removal of brain lesion | 5 | 14 | 79% |
| 18 | 61542 | Removal of brain tissue | 5 | 7 | 80% |
| 19 | 61781 | Scan proc cranial intra | 5 | 187 | 81% |
| 20 | 61120 | Burr hole for puncture | 4 | 9 | 82% |
| 21 | 61343 | Incise skull (press relief) | 4 | 9 | 83% |
| 22 | 61760 | Implant brain electrodes | 4 | 4 | 84% |
| 23 | 61050 | Remove brain canal fluid | 3 | 3 | 84% |
| 24 | 61305 | Open skull for exploration | 3 | 5 | 85% |
| 25 | 61322 | Decompressive craniotomy | 3 | 16 | 85% |
| 26 | 61516 | Removal of brain lesion | 3 | 6 | 86% |
| 27 | 61537 | Removal of brain tissue | 3 | 5 | 86% |
| 28 | 61539 | Removal of brain tissue | 3 | 3 | 87% |
| 29 | 61548 | Removal of pituitary gland | 3 | 3 | 87% |
| 30 | 61591 | Infratemporal approach/skull | 3 | 4 | 88% |
| 31 | 61783 | Scan proc spinal | 3 | 3 | 89% |
| 32 | 61154 | Pierce skull & remove clot | 2 | 6 | 89% |
| 33 | 61313 | Open skull for drainage | 2 | 21 | 89% |
| 34 | 61314 | Open skull for drainage | 2 | 2 | 90% |
| 35 | 61316 | Implt cran bone flap to abdo | 2 | 2 | 90% |
| 36 | 61321 | Open skull for drainage | 2 | 5 | 90% |
| 37 | 61323 | Decompressive lobectomy | 2 | 4 | 91% |
| 38 | 61501 | Remove infected skull bone | 2 | 3 | 91% |
| 39 | 61519 | Remove brain lining lesion | 2 | 4 | 92% |
| 40 | 61531 | Implant brain electrodes | 2 | 4 | 92% |
| 41 | 61533 | Implant brain electrodes | 2 | 4 | 92% |
| 42 | 61534 | Removal of brain lesion | 2 | 4 | 93% |
| 43 | 61583 | Craniofacial approach skull | 2 | 6 | 93% |
| 44 | 61590 | Infratemporal approach/skull | 2 | 3 | 93% |
| 45 | 61001 | Remove cranial cavity fluid | 1 | 1 | 94% |
| 46 | 61055 | Injection into brain canal | 1 | 1 | 94% |
| 47 | 61105 | Twist drill hole | 1 | 2 | 94% |
| 48 | 61108 | Drill skull for drainage | 1 | 1 | 94% |
| 49 | 61156 | Pierce skull for drainage | 1 | 2 | 94% |
| 50 | 61215 | Insert brain-fluid device | 1 | 4 | 95% |
| 51 | 61315 | Open skull for drainage | 1 | 2 | 95% |
| 52 | 61330 | Decompress eye socket | 1 | 9 | 95% |
| 53 | 61340 | Subtemporal decompression | 1 | 1 | 95% |
| 54 | 61345 | Relieve cranial pressure | 1 | 6 | 95% |
| 55 | 61458 | Incise skull for brain wound | 1 | 1 | 96% |
| 56 | 61490 | Incise skull for surgery | 1 | 1 | 96% |
| 57 | 61500 | Removal of skull lesion | 1 | 1 | 96% |
| 58 | 61520 | Removal of brain lesion | 1 | 1 | 96% |
| 59 | 61536 | Removal of brain lesion | 1 | 2 | 96% |
| 60 | 61538 | Removal of brain tissue | 1 | 1 | 96% |
| 61 | 61541 | Incision of brain tissue | 1 | 1 | 97% |
| 62 | 61543 | Removal of brain tissue | 1 | 1 | 97% |
| 63 | 61571 | Incise skull for brain wound | 1 | 2 | 97% |
| 64 | 61575 | Skull base/brainstem surgery | 1 | 1 | 97% |
| 65 | 61580 | Craniofacial approach skull | 1 | 5 | 97% |
| 66 | 61585 | Orbitocranial approach/skull | 1 | 7 | 98% |
| 67 | 61586 | Resect nasopharynx skull | 1 | 6 | 98% |
| 68 | 61592 | Orbitocranial approach/skull | 1 | 2 | 98% |
| 69 | 61600 | Resect/excise cranial lesion | 1 | 10 | 98% |
| 70 | 61615 | Resect/excise lesion skull | 1 | 3 | 98% |
| 71 | 61619 | Repair dura | 1 | 14 | 99% |
| 72 | 61630 | Intracranial angioplasty | 1 | 1 | 99% |
| 73 | 61680 | Intracranial vessel surgery | 1 | 2 | 99% |
| 74 | 61697 | Brain aneurysm repr complx | 1 | 2 | 99% |
| 75 | 61698 | Brain aneurysm repr complx | 1 | 1 | 99% |
| 76 | 61700 | Brain aneurysm repr simple | 1 | 1 | 99% |
| 77 | 61711 | Fusion of skull arteries | 1 | 1 | 100% |
| 78 | 61795 | Brain surgery using computer | 1 | 29 | 100% |
| 79 | 61880 | Revise/remove neuroelectrode | 1 | 2 | 100% |
| 80 | 61000 | Remove cranial cavity fluid | 0 | 1 | 100% |
| 81 | 61026 | Injection into brain canal | 0 | 1 | 100% |
| 82 | 61151 | Pierce skull for drainage | 0 | 1 | 100% |
| 83 | 61250 | Pierce skull & explore | 0 | 0 | 100% |
| 84 | 61253 | Pierce skull & explore | 0 | 0 | 100% |
| 85 | 61332 | Explore/biopsy eye socket | 0 | 0 | 100% |
| 86 | 61333 | Explore orbit/remove lesion | 0 | 1 | 100% |
| 87 | 61440 | Incise skull for surgery | 0 | 0 | 100% |
| 88 | 61460 | Incise skull for surgery | 0 | 0 | 100% |
| 89 | 61517 | Implt brain chemotx add-on | 0 | 1 | 100% |
| 90 | 61521 | Removal of brain lesion | 0 | 0 | 100% |
| 91 | 61522 | Removal of brain abscess | 0 | 0 | 100% |
| 92 | 61524 | Removal of brain lesion | 0 | 2 | 100% |
| 93 | 61526 | Removal of brain lesion | 0 | 0 | 100% |
| 94 | 61530 | Removal of brain lesion | 0 | 0 | 100% |
| 95 | 61535 | Remove brain electrodes | 0 | 1 | 100% |
| 96 | 61540 | Removal of brain tissue | 0 | 1 | 100% |
| 97 | 61544 | Remove & treat brain lesion | 0 | 0 | 100% |
| 98 | 61545 | Excision of brain tumor | 0 | 0 | 100% |
| 99 | 61546 | Removal of pituitary gland | 0 | 0 | 100% |
| 100 | 61556 | Incise skull/sutures | 0 | 1 | 100% |
| 101 | 61557 | Incise skull/sutures | 0 | 0 | 100% |
| 102 | 61558 | Excision of skull/sutures | 0 | 0 | 100% |
| 103 | 61559 | Excision of skull/sutures | 0 | 0 | 100% |
| 104 | 61566 | Removal of brain tissue | 0 | 1 | 100% |
| 105 | 61567 | Incision of brain tissue | 0 | 0 | 100% |
| 106 | 61570 | Remove foreign body brain | 0 | 0 | 100% |
| 107 | 61581 | Craniofacial approach skull | 0 | 2 | 100% |
| 108 | 61582 | Craniofacial approach skull | 0 | 0 | 100% |
| 109 | 61584 | Orbitocranial approach/skull | 0 | 0 | 100% |
| 110 | 61595 | Transtemporal approach/skull | 0 | 1 | 100% |
| 111 | 61596 | Transcochlear approach/skull | 0 | 0 | 100% |
| 112 | 61597 | Transcondylar approach/skull | 0 | 0 | 100% |
| 113 | 61598 | Transpetrosal approach/skull | 0 | 0 | 100% |
| 114 | 61601 | Resect/excise cranial lesion | 0 | 11 | 100% |
| 115 | 61605 | Resect/excise cranial lesion | 0 | 0 | 100% |
| 116 | 61606 | Resect/excise cranial lesion | 0 | 4 | 100% |
| 117 | 61607 | Resect/excise cranial lesion | 0 | 2 | 100% |
| 118 | 61608 | Resect/excise cranial lesion | 0 | 0 | 100% |
| 119 | 61616 | Resect/excise lesion skull | 0 | 1 | 100% |
| 120 | 61618 | Repair dura | 0 | 15 | 100% |
| 121 | 61623 | Endovasc tempory vessel occl | 0 | 0 | 100% |
| 122 | 61626 | Transcath occlusion non-cns | 0 | 0 | 100% |
| 123 | 61635 | Intracran angioplsty w/stent | 0 | 0 | 100% |
| 124 | 61640 | Dilate ic vasospasm init | 0 | 2 | 100% |
| 125 | 61641 | Dilate ic vasospasm add-on | 0 | 0 | 100% |
| 126 | 61642 | Dilate ic vasospasm add-on | 0 | 0 | 100% |
| 127 | 61682 | Intracranial vessel surgery | 0 | 0 | 100% |
| 128 | 61684 | Intracranial vessel surgery | 0 | 0 | 100% |
| 129 | 61686 | Intracranial vessel surgery | 0 | 0 | 100% |
| 130 | 61692 | Intracranial vessel surgery | 0 | 0 | 100% |
| 131 | 61702 | Inner skull vessel surgery | 0 | 0 | 100% |
| 132 | 61708 | Revise circulation to head | 0 | 0 | 100% |
| 133 | 61710 | Revise circulation to head | 0 | 0 | 100% |
| 134 | 61770 | Incise skull for treatment | 0 | 0 | 100% |
| 135 | 61796 | Srs cranial lesion simple | 0 | 1 | 100% |
| 136 | 61800 | Apply srs headframe add-on | 0 | 0 | 100% |
| 137 | 61860 | Implant neuroelectrodes | 0 | 0 | 100% |
| 138 | 61863 | Implant neuroelectrode | 0 | 0 | 100% |
| 139 | 61867 | Implant neuroelectrode | 0 | 0 | 100% |
| 140 | 61868 | Implant neuroelectrde addl | 0 | 0 | 100% |
| 141 | 61885 | Insrt/redo neurostim 1 array | 0 | 0 | 100% |
| 142 | 61886 | Implant neurostim arrays | 0 | 0 | 100% |
| 143 | 61888 | Revise/remove neuroreceiver | 0 | 0 | 100% |
| 144 | 62010 | Treatment of head injury | 0 | 0 | 100% |
| 145 | 62100 | Repair brain fluid leakage | 0 | 0 | 100% |
| 146 | 62115 | Reduction of skull defect | 0 | 0 | 100% |
| 147 | 62116 | Reduction of skull defect | 0 | 0 | 100% |
| 148 | 62120 | Repair skull cavity lesion | 0 | 0 | 100% |
| 149 | 62121 | Incise skull repair | 0 | 0 | 100% |
| 150 | 62140 | Repair of skull defect | 0 | 0 | 100% |
| 151 | 62141 | Repair of skull defect | 0 | 0 | 100% |
| 152 | 62142 | Remove skull plate/flap | 0 | 0 | 100% |
| 153 | 62143 | Replace skull plate/flap | 0 | 0 | 100% |
| 154 | 62145 | Repair of skull & brain | 0 | 0 | 100% |
| 155 | 62146 | Repair of skull with graft | 0 | 0 | 100% |
| 156 | 62147 | Repair of skull with graft | 0 | 0 | 100% |
| 157 | 62148 | Retr bone flap to fix skull | 0 | 0 | 100% |
| 158 | 62160 | Neuroendoscopy add-on | 0 | 0 | 100% |
| 159 | 62161 | Dissect brain w/scope | 0 | 0 | 100% |
| 160 | 62162 | Remove colloid cyst w/scope | 0 | 0 | 100% |
| 161 | 62163 | Zneuroendoscopy w/fb removal | 0 | 0 | 100% |
| 162 | 62164 | Remove brain tumor w/scope | 0 | 0 | 100% |
| 163 | 62165 | Remove pituit tumor w/scope | 0 | 0 | 100% |
| 164 | 62180 | Establish brain cavity shunt | 0 | 0 | 100% |
| 165 | 62190 | Establish brain cavity shunt | 0 | 0 | 100% |
| 166 | 62192 | Establish brain cavity shunt | 0 | 0 | 100% |
| 167 | 62194 | Replace/irrigate catheter | 0 | 0 | 100% |
| 168 | 62200 | Establish brain cavity shunt | 0 | 0 | 100% |
| 169 | 62201 | Brain cavity shunt w/scope | 0 | 0 | 100% |
| 170 | 62220 | Establish brain cavity shunt | 0 | 0 | 100% |
| 171 | 62223 | Establish brain cavity shunt | 0 | 0 | 100% |
| 172 | 62225 | Replace/irrigate catheter | 0 | 0 | 100% |
| 173 | 62230 | Replace/revise brain shunt | 0 | 0 | 100% |
| 174 | 62252 | Csf shunt reprogram | 0 | 0 | 100% |
| 175 | 62256 | Remove brain cavity shunt | 0 | 0 | 100% |
| 176 | 62258 | Replace brain cavity shunt | 0 | 0 | 100% |
| 177 | 62267 | Interdiscal perq aspir dx | 0 | 0 | 100% |
| 178 | 62272 | Drain cerebro spinal fluid | 0 | 0 | 100% |

Source: Authors’ Analysis of 2010 to 2014 Truven Health MarketScan Commercial Claims and Encounters Database

Table E shows the number of patients by combinations of the five patient subgroups:

- Subgroup 1: Patients who had a LOS greater than two days (N=16,226 meningitis and N=6,468 encephalitis)
- Subgroup 2: Patients who were admitted to an ICU any time during a hospital stay (N=5,894 meningitis and N=3,430 encephalitis)
- Subgroup 3: Patients who received a neurosurgical procedure (N=1,233 meningitis and N=535 encephalitis)
- Subgroup 4: Patients who had a diagnosis code indicating that they had HIV-1 infection or a previous organ transplant (N=513 meningitis and N=289 encephalitis)
- Subgroup 5: Patients who were less than one year old at the time of admission (N=2,835 meningitis and N=174 encephalitis).

Table E: Number of Patients by Patient Subgroup Combination

| Patient Subgroup(s) | Number of Patients Hospitalized with Meningitis | Number of Patients Hospitalized with Encephalitis |
| --- | --- | --- |
| 1 | 9,246 | 3,114 |
| 1,2 | 3,607 | 2,470 |
| 1,2,3 | 951 | 409 |
| 1,2,3,4 | 15 | 14 |
| 1,2,3,5 | 45 | 7 |
| 1,2,4 | 149 | 121 |
| 1,2,5 | 378 | 61 |
| 1,3 | 129 | 54 |
| 1,3,4 | 6 | 6 |
| 1,3,5 | 37 | 2 |
| 1,4 | 272 | 126 |
| 1,4,5 | 1 | 0 |
| 1,5 | 1,390 | 84 |
| 2 | 642 | 305 |
| 2,3 | 24 | 33 |
| 2,3,4 | 0 | 1 |
| 2,4 | 10 | 7 |
| 2,5 | 73 | 2 |
| 3 | 26 | 9 |
| 4 | 60 | 14 |
| 5 | 911 | 18 |
| No subgroup | 5,961 | 1,001 |
| Total | 23,933 | 7,858 |

Source: Authors’ analysis of 2010 to 2014 Truven Health MarketScan Commercial Claims and Encounters Database

# References

1. Nigrovic LE, Fine AM, Monuteaux MC, Shah SS, Neuman MI. Trends in the Management of Viral Meningitis at United States Children’s Hospitals. Pediatrics. 2013;131(4):670-6.

2. Takhar SS, Ting SA, Camargo CA, Pallin DJ. US Emergency Department Visits for Meningitis, 1993–2008. Acad Emerg Med. 2012;19(6):632-9.

3. Holmquist L, Russo CA, Elixhauser A. Meningitis-Related Hospitalizations in the United States. HCUP Statistical Brief #57. Rockville, MD: Agency for Healthcare Research and Quality; 2008.

4. Vora NM, Holman RC, Mehal JM, Steiner CA, Blanton J, Sejvar J. Burden of Encephalitis-Associated Hospitalizations in the United States, 1998–2010. Neurology. 2014 February 4, 2014;82(5):443-51.
